# Attitudes and beliefs among patients treated with mood stabilizers

**DOI:** 10.1186/1745-0179-2-8

**Published:** 2006-05-19

**Authors:** Lars Vedel Kessing, Hanne Vibe Hansen, Per Bech

**Affiliations:** 1 Department of Psychiatry, Rigshospitalet, University Hospital of Copenhagen, Copenhagen, Denmark; 2 Psychiatric Research Unit, Frederiksborg General Hospital, Hillerød, Denmark

## Abstract

**Background:**

There is increasing evidence that attitudes and beliefs are important in predicting adherence in depressive and bipolar disorders. However, such attitudes and beliefs on mood stabilizers have not been analysed by socio-demographic and clinical variables.

**Methods:**

The Mood Stabilizer Compliance Questionnaire (MSQC) was mailed to a large population of patients with depressive or bipolar disorder representative of patients treated at their first contacts to hospital settings in Denmark.

**Results:**

Of the 1005 recipients, 49.9 % responded to the letter and among these 256 indicated that they previously had been or currently were in treatment with a mood stabilizer. A large proportion of the patients (40 to 80 %) had non-correct views on the effect of mood stabilizers. Older patients consistently had a more negative view on the doctor-patient relationship, more non-correct views on the effect of mood stabilizers and a more negative view on mood stabilizers. There was no difference in the attitudes and beliefs according to the type of disorder (depressive or bipolar), the number of psychiatric hospitalisations or according to the type of the current doctor (general practitioner, private psychiatrist, community psychiatry doctor, hospital doctor, other doctor).

**Conclusion:**

There is a need of improving knowledge and attitudes toward diagnosis and treatment especially among elder patients as this may add to improve the prognosis of depressive and bipolar disorders.

## 1. Introduction

Medication non-adherence for depressive and bipolar disorders range from 10 to 60 % (median 40 %). It has recently been concluded in a review [1] that it seems as attitudes and beliefs are at least as important as side-effects in predicting adherence in depressive and bipolar disorders [2-4]. Beliefs and expectations has in several studies been found to be associated with non-adherence to lithium [1,5,6] and a recent study similarly concluded that attitudes and behaviours are better predictors of non-adherence to mood stabilizers than side effects of medication [7]. Nevertheless, most of the studies have included a rather small number of patients [5,8-11] which might explain the very few attempts that have been made to correlate attitudes and beliefs with gender and age or with diagnostic subtypes (unipolar versus bipolar disorder) [12].

Demyttenaere et al [13] have recently developed a questionnaire (The Antidepressants Compliance Questionnaire (ADQC)) to measure attitudes and beliefs concerning depression and antidepressive treatment. Factor analysis identified four meaningful components [13] and the test-retest reliability of the ADQC was found acceptable [13]. We have developed a questionnaire analogue to the ADQC to measure attitudes and beliefs concerning depressive and/or manic episodes and treatment with mood stabilizers, the Mood Stabilizer Compliance Questionnaire (MSCQ). We have thus replaced all the items concerning antidepressants with mood stabilizers and replaced items concerning depressive episodes with depressive and/or manic episodes. Apart from these adjustments no other changes have been made. Hence, the scoring of the items in the MSCQ is identical to the ADQC.

The objectives of the present study were (a) to characterise attitudes and beliefs on diagnosis and treatment with mood stabilizers by means of the MSQC among patients with severe depressive and bipolar disorders treated at their first contacts to hospital psychiatry and (b) to analyse the results in relation to socio-demographic and clinical variables. Thus, we have investigated the score on the MSQC in relation to age and gender and tested whether the score was different for patients with depressive disorder and patients with bipolar disorder and whether the score was related to the number of severe affective episodes patients had experienced.

## 2. Methods

### 2.1. The register

The Danish Psychiatric Central Research Register (DPCRR) is nation-wide with registration of all psychiatric hospitalisations in Denmark for the 5.3 million inhabitants [14]. From January 1, 1995 the register included information on patients in psychiatric ambulatories and community psychiatric centres, also.

All inhabitants in Denmark have a unique person identification number (Civil Person Registration number, CPR-number) that can be logically checked for errors; so it can be established with great certainty if a patient has had contact to psychiatric service previously, irrespective of changes in name etc. The International Classification of Diseases, 10th Revision [15] has been used in Denmark from January 1, 1994.

### 2.2. The sample

A total sample of 1005 patients with address in Denmark was identified as follows by means of the Danish Psychiatric Central Research Register:

1. A random sample including 25 % of all patients who in 2002, at their *first discharge ever *from a psychiatric hospital, had received the diagnosis of single or recurrent depression (ICD-10, DF32-33). N = 311.

2. All patients who in 2002, at their *third discharge ever *from a psychiatric hospital, had received a diagnosis of recurrent depression (ICD-10, DF33). N = 213.

3. All patients who in 2002, at their *first discharge ever *from a psychiatric hospital, had received a diagnosis of mania/bipolar affective disorder (ICD-10, DF30-31). N = 181.

4. All patients who in 2002, at their *third discharge ever *from psychiatric hospital, had received a diagnosis of bipolar affective disorder (ICD-10, DF31). N = 195.

5. All patients who in 2001 or 2002, at their *first outpatient visit ever *to a psychiatric ambulatory or community-psychiatric centre, had received a diagnosis of mania/bipolar affective disorder (ICD-10, DF30-31). N = 105.

Patients were included in this way to get a greater variation in the number of admissions.

2.3. The Mood Stabilizer Compliance Questionnaire (MSQC)

The Mood Stabilizer Compliance Questionnaire (MSQC) is developed as an analogue questionnaire to the Antidepressant Compliance Questionnaire (ADCQ) by Demyttenaere [13] with 33 analogue questions.

MSQC consists of 33 items that are scored as follows: 1 (mostly disagree), 2 (rather disagree), 3 (rather agree) and 4 (mostly agree) [13]. Mean values (SD) were calculated according to the ADCQ scoring system from 1 to 4 by Demyttenaere [13]. The higher the score, the more positive the patients beliefs and attitudes toward compliance are. The 33 items were subdivided into four components of items: component 1 (perceived doctor-patient relationship, items 2, 5, 7, 12, 14, 15, 16, 19, 20, 21, 22, 25, 27, 28, 29), component 2 (beliefs on mood stabilizers, items 4, 10, 11, 17, 18, 32, 33), component 3 (preserved autonomy in general, items 1, 3, 6, 9, 13, 24) and component 4 (preserved autonomy in dosing of mood stabilizers, items 8, 23, 26, 30) in a way nearly similar to the ADCQ [13].

The MSQC was mailed to patients as part of a larger survey during spring 2004. The study was carried out in compliance with the Helsinki Declaration. The local ethical committee approved the study (KF 01-159/02) allowing mailing of one reminder only if patients did not respond to the initial letter.

### 2.4. Statistical analysis

In univariate analyses, categorical data were analysed with chi-square test (2-sided) and continuous data were analysed with the Mann-Whitney test for two independent groups. In multiple regression analyses, component 1, 2, 3, 4 of the MSQC were included as outcome, respectively, and gender, age at first contact, number of admissions and type of disorder (depressive versus bipolar disorder) were included as predictive variables.

P < 0.05 was used to indicate statistical significance. SPSS software package for windows, version 11.0 was used [16].

## 3. Results

Among the 1005 patients who were identified in the register with a diagnosis of depressive disorder or mania/bipolar disorder, 16 patients were excluded (7 due to unknown address, 6 as the patients did not understand Danish, 2 as the patients according to relatives were to demented and 1 as the patient has died). Among the remaining 989 patients who were potentially able to respond to the questionnaires, 493 patients fulfilled the questionnaires, corresponding to a response rate of 49.9 %. Among the 493 patients who fulfilled the questionnaires, 256 (51.9 %) responded that they previously or currently were in treatment with a mood stabilizer. Totally, 108 patients indicated that they currently were taking lithium, 10 patients were taking carbamazepine, 14 patient valproate and 35 patients were currently taking lamotrigene. Among these patients, 7 patients got combinations of various kinds of mood stabilizers. We did not ask for the type of the prior mood stabilizers, as such data may be inaccurate. As can be seen from Table 1, significantly more women (53.4 %) than men (45.2 %) responded to the overall questionnaires (p = 0.006). No significant age differences at first discharge were seen between the patients with depressive and those with bipolar disorder or between the numbers of admissions in responders versus non-responders to the questionnaire.

**Table 1:** Socio-demographic and clinical characteristics of responders and non-responders to the questionnaires.

	Responders N = 493	Non-responders N = 496	P
Age (median (quartiles))	43.8 (33.3–53.4)	45.5 (33.5–55.8)	0.2
Men (%)	194 (45.2)	235 (54.8)	0.006
Women (%)	299 (53.4)	261 (46.6)	
Depressive disorder (%)	258 (50.0)	258 (50.0)	0.9
Bipolar disorder (%)	235 (49.7)	238 (50.3)	
Number of admissions (SD)	2.44 (2.24)	2.38 (1.42)	0.8

The responses to the 33 items of the MSQC are presented in Table 2 for the 256 patients who reported that they previously or currently were in treatment with a mood stabilizer. Mean (SD) values for each item are presented for patients with depressive and bipolar disorder, separately (also for comparison with future studies). Mean values were calculated according to the scoring system from 1 to 4 by Demyttenaere [13]. The higher the score, the more positive the patients beliefs and attitudes toward compliance are. There were statistical differences in the scores between depressive and bipolar disorder in four items (items 4, 5, 18 and 24).

**Table 2:** Frequency distribution (%) of the 33 items of MSCQ. N = 256.

		Mostly disagree %	Rather disagree %	Rather agree %	Mostly agree %	Depressive Mean (SD)	Bipolar Mean (SD)
1	As long as you are taking mood stabilizers you do not really know if they are actually necessary	11.5	11.1	23.8	53.0	1.82 (1.02)	2.00 (1.16)
2	My doctor listens properly to what I think about mood stabilizers	5.9	7.1	30.3	56.7	1.52 (0.95)	1.53 (0.80)
3	When you have taken mood stabilizers over a long period of time it is difficult to stop taking them	31.0	19.0	22.7	27.3	2.64 (1.14)	2.69 (1.14)
4	With mood stabilizers my depressions and/or manic episodes disappear	15.3	12.5	40.3	31.9	2.57 (1.15)	1.88 (0.85)*
5	My doctor has made me feel confident that mood stabilizers are the suitable treatment for me	9.5	8.7	28.1	53.7	1.93 (1.11)	1.59 (0.90)*
6	When you take mood stabilizers you have less control over your thoughts and feelings	43.9	18.9	21.3	16.0	3.09 (1.05)	2.98 (1.08)
7	My doctor takes sufficient time to listen to my problems	7.3	6.1	25.7	60.8	1.57 (0.97)	1.48 (0.69)
8	You may take fewer tablets than prescribed on days when you feel better	86.2	5.7	4.5	3.6	3.77 (0.71)	3.79 (0.61)
9	Mood stabilizers can alter your personality	39.3	19.0	24.0	17.8	2.82 (1.17)	2.90 (1.09)
10	My partner agrees that mood stabilizers are a suitable treatment for my condition	7.1	5.9	25.4	61.5	1.75 (0.97)	1.45 (0.79)
11	Mood stabilizers correct the changes that occurred in my brain due to stress or problems	13.7	14.9	34.9	36.5	2.20 (1.05)	1.95 (1.08)
12	My doctor has explained the causes of my disorder sufficiently	18.9	12.9	24.9	43.3	2.00 (1.18)	1.96 (1.11)
13	Your body can become addicted to mood stabilizers	30.8	19.4	16.9	32.9	2.50 (1.29)	2.61 (1.16)
14	My doctor takes sufficient time to discuss my emotional problems	8.7	12.4	26.6	52.3	1.61 (0.92)	1.76 (0.93)
15	My doctor has explained depression and mania sufficiently to me	12.9	11.2	25.8	50.2	1.89 (1.19)	1.81 (1.01)
16	My doctor shows sufficient consideration for my views and feelings about his treatment with mood stabilizers	8.8	7.1	30.8	53.3	1.82 (1.11)	1.64 (0.85)
17	Mood stabilizers help me to worry less about my problems	13.4	15.9	33.7	37.0	2.11 (0.95)	2.10 (1.086)
18	My partner agrees that depressive disorder or bipolar disorder is the correct diagnosis of my condition	16.0	8.9	21.9	53.3	2.34 (1.29)	1.60 (0.89)*
19	I receive sufficient psychological support and encouragement from my doctor	9.2	10.0	28.9	51.9	1.64 (0.97)	1.66 (0.84)
20	My doctor fully understands my condition	7.4	10.3	21.9	60.3	1.55 (0.975)	1.69 (0.92)
21	My doctor strongly emphasises that it is important to take the mood stabilizers regularly	5.7	3.7	20.5	70.1	1.52 (0.98)	1.35 (0.77)
22	My doctor is really interested in my problems	6.6	7.5	22.8	63.1	1.50 (1.00)	1.51 (0.83)
23	If you forget to take the mood stabilizer on a certain day, it is better to take an additional dose the following day	81.6	7.8	6.5	4.1	3.75 (0.69)	3.64 (0.82)
24	Your body can become immune to mood stabilizers	42.3	21.6	19.4	16.7	2.64 (1.16)	3.05 (1.08)*
25	My doctor listens properly when I tell him what it is like to be depressed	9.2	10.5	28.5	51.8	1.70 (1.05)	1.70 (0.88)
26	You may take more tablets than prescribed on days when you feel more depressed	87.1	6.2	2.5	4.1	3.80 (0.55)	3.85 (0.51)
27	My doctor understands my feelings and thoughts in depression and mania perfectly	11.3	11.8	30.7	46.2	1.66 (1.01)	1.88 (0.96)
28	My doctor has explained properly about mood stabilizers, their action and side effects	11.5	11.1	28.0	49.4	1.82 (1.04)	1.75 (1.01)
29	My doctor listens properly to what I consider to be the causes of my depression and/or manias	10.1	8.8	32.5	48.7	1.70 (1.00)	1.78 (0.93)
30	Skipping a day now and again prevents your body from becoming immune to the mood stabilizers	87.5	9.2	2.1	1.3	3.84 (0.53)	3.83 (0.47)
31	I think my depression and/or manic episodes are mainly due to factors associated with my personality	20.3	18.6	34.2	26.8	2.36 (1.14)	2.23 (1.06)
32	My emotional problems are solved by the mood stabilizers	27.5	24.2	30.8	17.5	2.77 (1.05)	2.61 (1.10)
33	Mood stabilizers make me stronger so I will be able to deal more efficiently with my problems	12.8	12.8	39.5	35.0	2.11 (1.02)	1.91 (0.98)

* In items 4, 5, 18 and 24 there are statistically significant differences between depressive and bipolar disorder (P ≤ 0.05). For all other items P > 0.05 in comparisons of depressive and bipolar disorder.

In general, the major proportion of patients agreed on the diagnosis and the choice and effect of pharmacological treatment and the majority felt content with their doctor and with information regarding diagnosis and treatment. Only minorities of patients had wrong ideas about dosing or the effect of mood stabilizers (You may take fewer tablets than prescribed on days when you feel better (8.1 %, item 8). If you forget to take the mood stabilizer on a certain day, it is better to take an additional dose the following day (10.6 %, item 23). You may take more tablets than prescribed on days when you feel more depressed (6.6 %, item 26). Skipping a day now and again prevents your body from becoming immune to the mood stabilizers (3.4 %, item 30)).

In contrast, a large proportion of the patients had non-correct views on the effect of mood stabilizers (Component2 (Preserved autonomy)). A total of 77.4 % of the sample of patients agreed on item 1 that as long as you are taking mood stabilizers you do not really know if they are actually necessary. Accordingly, 50.0 % agreed on item 3 that when you have taken mood stabilizers over a long period of time it is difficult to stop taking them and 37.3 % agreed on item 6 that when you take mood stabilizers you have less control over your thoughts and feelings. Further, 41.7 % agreed that mood stabilizers can alter your personality (item 9) and 49.8 % that your body can become addicted to mood stabilizers (item 13) and accordingly 36.1 % agreed that your body can become immune to mood stabilizers (item 24). A total of 61.0 % agreed that their depression and/or manic episodes are mainly due to factors associated with their personality (item 31).

Table 3 presents results from multiple regression analyses with component 1, 2, 3, 4 as outcome, respectively, and with inclusion of gender, age at first contact, number of admissions and type of disorder (depressive versus bipolar disorder) as predictive variables. As can be seen, age at first contact was negatively associated with all four components (higher age associated with lower score), however not significantly with component 4 (*B *= -0.003, p = 0.2). There were no significant associations between any component and gender, number of admissions or type of disorder (depressive disorder versus bipolar disorder). Including further a variable of educational level (dichotomised as primary school versus high school) did not change the results substantially, although age only was marginally associated with component 3 in this model (*B *= -0.009, p = 0.07). In these models, educational level was significantly and positively associated with component 3, only (*B *= 0.40, p = 0.002).

**Table 3:** Age at first contact, gender, number of admissions and type of disorder (depressive or bipolar disorder) in relation to the values of the four components of the Mood Stabilizer Compliance Questionnaire.

	Perceived doctor-patient relationship Component 1	Positive beliefs on Mood stabilizers Component 2	Preserved Autonomy: general Component 3	Preserved Autonomy: dosing of mood stabilizers Component 4
	
	*B *(95 % CI)	*B *(95 % CI)	*B *(95 % CI)	*B *(95 % CI)
Age	-0.010 (-0.02 – 0.004)**	-0.010 (-0.02 – 0.005)**	-0.015 (-0.02 – 0.005)**	-0.003 (-0.009–0.002)
Female gender	-0.19 (-0.41–0.03)	-0.01 (-0.23–0.20)	0.14 (-0.11–0.38)	-0.03 (-0.11–0.16)
Number of admissions	-0.006 (-0.076–0.064)	-0.008 (-0.08–0.06)	0.003 (-0.06–0.08)	0.007 (-0.04–0.05)
Depressive versus bipolar disorder	-0.18 (-0.41–0.04)	-0.20 (-0.42–0.02)	0.02 (-0.22–0.26)	-0.03 (-0.17–0.10)

* P ≤ 0.05 ** P < 0.01

All variables were included at the same time in the multiple regression models.

The above mentioned multiple regression models were repeated including further the variable describing the type of current doctor during outpatient treatment (general practitioner, private psychiatrist, community psychiatry doctor, hospital doctor, other doctor). There were no significant associations between any of the scores and type of doctor (p > 0.05).

## 4. Discussion

The most consistent finding in the study was that among patients discharged from hospital with depressive or bipolar disorders, older patients consistently had a more negative view on the doctor-patient relationship (Component 1), a more negative view on mood stabilizers (Component 2) and more non-correct views on the effect of mood stabilizers (Component 3). Further, adjusting for differences in age, there were no differences between patients with depressive and patients with bipolar disorder in any component. There was no difference in the attitudes and beliefs according to the number of psychiatric hospitalisations or according to the type of the current doctor (general practitioner, private psychiatrist, community psychiatry doctor, hospital doctor, other doctor).

Although the major proportion of patients with depressive disorder and as well as with bipolar disorder agreed on the diagnosis and the choice and effect of pharmacological treatment and the majority felt content with their doctor and with information a large proportion of the patients had non-correct views on the effect of mood stabilizers (Component 3 and 4 (Preserved autonomy)). A total of 77.4 % of patients believed that as long as you are taking a mood stabilizer you do not really know if they are actually necessary and 36 to 50 % that you can become addicted or that mood stabilizers can alter your personality. It is most probably that such attitudes may result in reluctance to take mood stabilizers in the long run.

We included questions on reasons for discontinuing treatment but to few patients answered these questions. It is well known that direct questions on reasons for stopping treatment is flawed by a low response rate and by low validity of the answers. Thus, there is no single valid way to measure non-adherence [17]. Questionnaires on attitudes and beliefs concerning illness and treatment such as the ADCQ and the MSCQ may be less provocative for patients and with a higher response rate.

We have previously reported on finding on attitudes and beliefs on antidepressants among the larger population of patients who received antidepressants [18]. Thus, among the 493 patients who participated in the survey, 422 reported that they previously or currently were in treatment with an antidepressant (and these patients fulfilled the ADCQ, [18]) and 256 that they previously or currently were in treatment with a mood stabilizer (and these patients fulfilled the MSCQ). The main proportion (88 %) of patients who previously or currently were in treatment with a mood stabilizer had at one point in time received an antidepressant and these patients fulfilled both questionnaires. The findings on attitudes and beliefs on mood stabilizers are surprisingly similar to the findings on attitudes and beliefs on antidepressants. It seems as patients who have negative attitudes toward mood stabilizers also have negative attitudes toward antidepressants as well, indicating a negative attitude toward medication in general.

It should be noted that patients who participated in the present study had had contact to the psychiatric health care system all over Denmark representing not only specialist university centres but also rural psychiatric hospitals, wards and centres.

Our findings are surprisingly similar to the results in a study by Schaub et al. who included a relatively small sample of patients (31 patients with depressive disorder and 74 with bipolar disorder) from a specialized lithium clinic [12]. In this study, patient's knowledge on lithium treatment correlated negatively with age, whereas sex, diagnosis and duration of treatment were unrelated to knowledge. The MSQC has a broader scope than focusing on knowledge of medication only, and further age was consistent related to a more negative view on the diagnosis and on the doctor-patient relationship. The relation to age is further in accordance with results from studies of the general population [19,20]: the elderly had less correct knowledge on mental illnesses and depression and were more critical toward diagnosis and treatment. However, as stated by Schaub et al. [12] associations about treatment-related knowledge have not often been examined formally in psychiatric settings, nor in settings of somatic diseases [21]. As mood stabilizers often are prescribed for many years and as elderly patients may present with cognitive dysfunction and somatic co-morbidity, lack of knowledge and a critical attitude toward diagnosis and treatment may add to deteriorate the prognosis even more. Such findings are consistent with the results from an ongoing pharmacoepidemiological study from our group showing that adherence to lithium is poor especially among patients aged 60 or more [22]. All together these findings are consistent with the findings in some studies that older patients with bipolar disorder may present more often with mixed episodes and have a lower treatment response [23] and a higher risk of recurrence [24].

Although seldom investigated, prior studies have not found gender differences in knowledge of treatment [12]. Similarly in the present study, no gender differences were found in attitudes toward diagnosis or treatment.

In the study by Schaub et al from a specialized lithium clinic, patients with depressive disorder had a lower knowledge about lithium treatment than patients with bipolar disorder [12]. In the present study there was no difference among patients with these disorders. ICD-10 does not discriminate between bipolar disorder type 1 and 2 as both are categorised as bipolar disorder [15]. We cannot exclude that some patients may have been misclassified as suffering from depressive disorder instead of bipolar disorder and that this may have diluted possible differences between the two illnesses.

We did not find any associations between scores on MSQC and the number of psychiatric hospitalisations. We are not aware of any other study besides the study by Schaub et al [12] that has investigated the relation between course of illness and attitudes and beliefs on illness and treatment. To avoid bias such studies have to be conducted prospectively as discussed in the following section on caveats of the present study.

### 4.1. Caveats of the study

General pitfalls in relation to the study design has been reported elsewhere [18]. In short, overall, approximately 50 % of patients answered the letter, a rate that equals the response rate in satisfaction questionnaire surveys in general [25]. Contrary to most studies on satisfaction conducted by mail we did not exclude very ill patients, patients who had been involuntary hospitalised, patients with dementia, the illiterate or emigrants, etc [25]. The questionnaires were mailed to all patients who gained contact to psychiatric hospital health care as described in the inclusion criteria. Patients who responded to the MSQC had a rather positive view upon illness and their doctor-patient relationship so we cannot exclude that patients with a more negative attitude has responded in a less degree. However, we do not believe that this has caused major bias in the revealed associations between attitudes/beliefs and socio-demographic/clinical variables as only slightly more women responded to the questionnaires and as the responses did not vary according to age, type of disorder or number of hospitalisations (Table 1). Although there was no difference in the response rate among patients with a poor versus better course of illness as indicated by many versus few hospitalisations we cannot exclude that the prevalence of other indicators of a poor outcome, such as co-morbidity with substance abuse or personality disorders, was higher among non-responders.

The diagnoses were made by psychiatrist all over Denmark according to ICD-10 and was not standardised for research purposes.

We do not have information on the number of affective episodes patients have experienced. It is well known that patients are hospitalised for the most severe depressive episodes only (mainly with somatic or psychotic symptoms) and for moderate to severe manic episodes [26]. We cannot exclude that there exist an association between the number of episodes or the duration of the illnesses and attitudes and beliefs according on depression and mood stabilizers. However, using the number of psychiatric hospitalisations as a measure of the course of illness we did not find that patients with a more severe course of illness (i.e., more hospitalisations) had a more negative attitudes and beliefs. This negative finding may be due to bias as those patients who seek hospitalisations many times also may constitute the proportion of patients with severe illness with a more positive view upon treatment and the health care system.

Thus, the study presented cross sectional data. We plan to conduct a follow-up study analysing whether these cross sectional data on attitudes and beliefs on mood stabilizers predict the risk of hospitalisation in the future.

Finally, the study does not include data on adherence to treatment. Future studies may investigate whether the MSQC is predictive of compliance with mood stabilizers.

In conclusion, the study showed that there seems to be a lack of knowledge and a critical attitude toward diagnosis and treatment in patients treated with mood stabilizers and especially among the elderly patients. This may add to deteriorate the prognosis of depressive and bipolar disorders. There is a need to further investigate whether this is actually the case.

## Competing interests

The author(s) declare that they have no competing interests.
